# The Many Facets of Sphingolipids in the Specific Phases of Acute Inflammatory Response

**DOI:** 10.1155/2018/5378284

**Published:** 2018-02-06

**Authors:** Sabine Grösch, Alice V. Alessenko, Elisabetta Albi

**Affiliations:** ^1^University Hospital Frankfurt, Instiute of Clinical Pharmacology, Frankfurt/Main, Germany; ^2^Emanuel Institute of Biochemical Physics, Russian Academy of Sciences, Moscow, Russia; ^3^Department of Pharmaceutical Science, University of Perugia, Perugia, Italy

## Abstract

This review provides an overview on components of the sphingolipid superfamily, on their localization and metabolism. Information about the sphingolipid biological activity in cell physiopathology is given. Recent studies highlight the role of sphingolipids in inflammatory process. We summarize the emerging data that support the different roles of the sphingolipid members in specific phases of inflammation: (1) migration of immune cells, (2) recognition of exogenous agents, and (3) activation/differentiation of immune cells.

## 1. Introduction

### 1.1. What Are Sphingolipids, How They Are Metabolized, and Where They Are Located?

**Figure 1:**
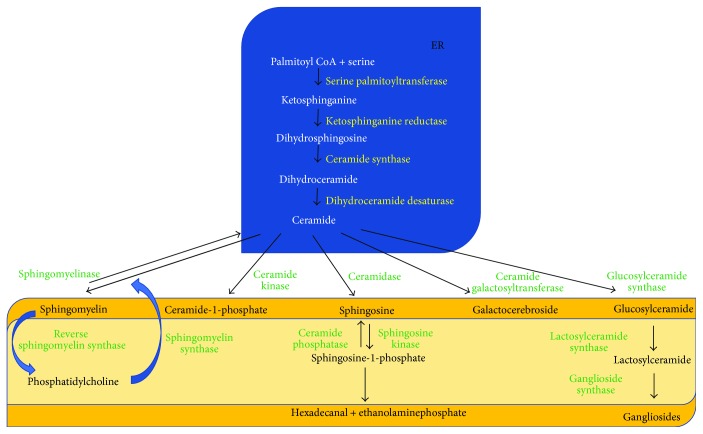
Sphingolipid pathways. The de novo synthesis occurs in endoplasmic reticulum (ER). Other biochemical pathways occur in the plasma, lysosome, and nucleus membranes.

Sphingolipids are an important class of lipids that play fundamental roles in cell life. The main sphingolipids include sphingomyelin (SM), ceramide (Cer), ceramide-1-phosphate (C1P), sphingosine (Sph), sphingosine-1-phosphate (S1P), glucosylceramide (GluCer), lactosylceramide (LacCer), gangliosides, and galactocerebrosides. All sphingolipids are metabolically interconnected in the equilibrium within the cells; each of them is rapidly produced to be used as a structural molecule and/or as a lipid mediator in response to a stimulus based on cellular needs. The metabolic pathway of sphingolipids includes (1) the de novo sphingolipid biosynthesis pathway with all intermediate bioactive molecules and (2) the SM catabolic pathway with all intermediate and final bioactive molecules (Figure 1).

SM is one of the most abundant sphingolipids in mammalian cell membranes. De novo synthesis of sphingolipids starts by the action of serine palmitoyltransferase that transfers the palmitic fatty acid to serine to form ketosphinganine that by ketosphinganine reductase is transformed into dihydroSph (also known as sphinganine). The dihydroSph is N-acetylated by ceramide synthases (CerS) that exists in 6 isoforms (CerS1 to CerS6), which add fatty acyl chains of defined chain length to dihydroSph to generate dihydroCer. DihydroCer is converted to Cer by the action of dihydroCer desaturase. Cer can be converted by the glucosylceramide synthase (GluCer-synthase) to GluCer and further to LacCer by the action of lactosylceramide synthase (LacCer-synthase). The complex glycosylated ceramides are generated by different glycosyltransferases which are specific to sugar residues that they transfer to generate gangliosides. In addition, Cer can be converted by the Cer galactosyltransferase into galactocerebroside. Cer is also a precursor for the synthesis of SM by the SM-synthase that exists in 2 isoforms SM-synthase 1 and SM-synthase 2, by adding to Cer the phosphorylcholine (PPC) of phosphatidylcholine (PC) [1]. In the *salvage pathway*, SM is hydrolysed by the sphingomyelinase (SMase) to PPC and Cer [1]. SMases are distinguished on the basis of their optimal pH and Km values in neutral (n-SMase), acid (a-SMase), and alkaline (alk-SMase) sphingomyelinase [2]. n-SMase is responsible for the degradation of SM of cell membranes and cytosol, a-SMase in lysosomes, and alk-SMase at intranuclear level [2, 3]. SM can also be used to synthesize PC as the donor of PPC that is added to diacylglycerol by the reverse sphingomyelin synthase (RSM-synthase) [3]. Cer generated by the SMases can be either degraded to Sph and free fatty acids by the ceramidases [4] or directly converted to C1P by the Cer kinase (CerK) [5, 6]. C1P is generated at the inner plasma membrane of cells and transported to different intracellular compartments by the human lipid transfer protein CPTP (ceramide-1-phosphate transfer protein) [7]. In the plasma membranes, Sph is formed by neutral ceramidase in the presence of divalent cations at neutral pH [8]. Five human ceramidase genes have been identified, including *ASAH1*, *ASAH2*, *ACER1*, *ACER2*, and *ACER3*, and their protein products are classified as the acid (ASAH1), neutral (ASAH2), and alkaline ceramidase (ACER1–3) subtypes according to their pH optimum for their catalytic activity [9]. Ceramidases have diverse functions depending on their subcellular location and the local pH [10]. Acid ceramidase is responsible for the degradation of Cer within lysosomes [11]. Sph generated by the ceramidases can be phosphorylated to S1P by sphingosine kinase (SphK) [12]. There are 2 isoenzymes of the enzyme, SphK1 and SphK2. SphK1 is the major enzyme responsible for S1P formation [13, 14]. SphK1 is distributed in the cytosol, and SphK2 is localized in the nuclei [15]. The reaction is reversible thanks to SphPh. S1P can be irreversibly broken down by S1P lyase (S1PL) to ethanolamine phosphate and hexadecenal.

Sphingolipids and their metabolizing enzymes are expressed in almost all tissues of the mammalian organism and are distributed in different structures of the cells. CerK is particularly expressed in the brain, kidney, and liver, and it is very low in the colon [16]. Free Sph is present in the liver [17], HL60 cells [18], neutrophils [19], membranes, and purified nuclei [20, 21]. S1P is expressed at a very low amount in fibroblasts [22].

#### 1.1.1. Sphingolipids in Lipid Rafts

In cell membranes, sphingolipids are associated with sterols to form specialized plasma membrane microdomains called lipid rafts that facilitate ligand-receptor interaction, cellular signal transduction, and membrane protein trafficking [23, 24]. At the intranuclear level, the lipid microdomains are rich in SM and cholesterol and n-SMase is associated to the inner nuclear membrane of the liver [25] and embryonic hippocampal cells [26].

### 1.2. Phases of Acute Inflammation and Mediators

Inflammation can arise as a response of the immune system to damage caused by foreign bodies and/or infectious, chemical, physical agents with the aim to protect the organism. The acute inflammatory response envisages a series of specific phases that requires the involvement of different cells and molecules [27]. It begins with transient and nonconstant vasoconstriction due to the release of catecholamines, serotonin, thromboxane A2, and prostacyclin by different cells followed by vasodilation due to the release of nitric oxide, bradykinin, histamine, and E and I series prostaglandins resulting in slow blood flow. An increase in vascular permeability allows granulocytes (neutrophils, eosinophils, and basophils) or mast cells, in relation to the stimulus that induced the inflammation, to interact with endothelium. The following sequence of events involves margination, rolling, adhesion, and transmigration of the immune cells to the damaged tissue to exercise their defense role. Circulating monocytes from the blood migrate to the inflamed tissue and transform into macrophages. Each phase requires a set of specific bioactive molecules [27]. For the resolution of inflammation, the following fundamental stages occur: reepithelization, angiogenesis, granulation tissue formation, and collagen deposition. If inflammation does not resolve, B-lymphocytes are transformed into plasma cells that produce antibodies against specific antigens of the exogenous agent that has caused the damage. However, inflammation could also be directed against autoantigens leading to an autoimmune response. In addition to the release of antibodies by B-lymphocytes, a hallmark of inflammation is the release of cytokines and chemokines by different cell types. Of particular importance in several inflammatory and autoimmune diseases is the cytokine tumor necrosis factor-*α* (TNF-*α*) produced by activated monocytes and macrophages [28, 29]. TNF-*α* has been characterized as a pleiotropic cytokine critical for cell trafficking and inflammation [30] and host defense against various pathogens [31–33]. It is associated with several autoimmune and inflammatory diseases, such as rheumatoid arthritis [34], septic shock [29], and inflammatory bowel diseases [35].

## 2. Sphingolipids in Cell Pathophysiology

Sphingolipids are fundamental molecules for cell life since they play both structural and functional roles either in cell membranes or in the nucleus. As actors in cell structure, sphingolipids influence the fluidity of the cell membrane [36], nuclear membrane [2], and nuclear matrix [37] and form lipid rafts, as reported above. Functionally, sphingolipids act as second messengers in various signaling pathways, for example, via the activation or inhibition of several kinases and phosphatases [38–46]. In particular, Sph is capable to induce GTP cyclohydrolase [47], to inhibit NADPH oxidase by preventing the translocation of 47-phox, a cytosolic component of the enzyme, to the membranes [48], to inhibit CTP:phosphocholine cytidylyltransferase [49], and to activate phospholipase D [50]. Activation of various plasma membrane receptors, such as the PDGFR [22, 51], the Fc*ε*RI, and Fc*γ*RI [52] as well as the C5aR [53], was found to rapidly increase intracellular S1P production through the stimulation of the SphK. Inhibition of SphK stimulation strongly reduced or even prevented cellular events such as receptor-stimulated DNA synthesis, Ca^2+^ mobilization, and vesicular trafficking. Interest in S1P focused recently on two distinct cellular actions of this lipid, namely, its function as an extracellular ligand, activating specific G protein-coupled receptors, and its role as an intracellular second messenger [54]. S1P acts through five specific receptors (S1P1, S1P2, S1P3, S1P4, and S1P5) [55–57]. Moreover, numerous publications demonstrate the ability of Sph [58–66] and S1P [67] to induce mobilization of Ca^2+^ from intracellular stores. Ca^2+^ seems to be an important regulator of CerK activity most likely by the interaction with calmodulin (CaM); the binding of CaM to CerK enhances CerK activity and the formation of C1P intracellular [65]. In this way, sphingolipids are now known to mediate cell proliferation [66, 68], differentiation [69], apoptosis [70, 71], stress response [72, 73], neuronal physiopathology [74], platelet aggregation [75], inhibition of blood coagulation [76], and cancer [77].

## 3. Roles of Sphingolipids in Specific Phases of Acute Inflammation

Sphingolipids have different roles in fundamental phases of the acute inflammatory response such as migration of immune cells, recognition of exogenous agents, and activation/differentiation of immune cells.

### 3.1. Migration of Immune Cells

**Figure 2:**
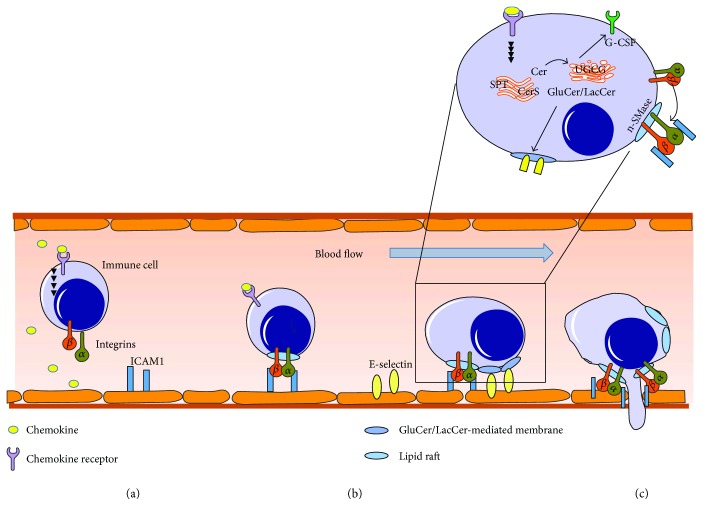
Activation, migration, and invasion of immune cells from the blood are influenced by several sphingolipids. (a) Initial adhesion step mediated by activation of immune cells by cytokines or chemokines and subsequent activation of integrins. (b) Activated integrins translocate into lipid rafts and bind to endothlial receptors like ICAM1. GluCer-enriched membranes are important for interaction with E-selectin. (c) Migration of immune cells is dependent on n-SMase and C1P.

The infiltration of immune cells into the sites of lesion and further their migration to proximate lymph nodes requires their exit from the blood stream and their migration across the basement membrane, a process that involves the interaction of selectins and subsequent integrins on immune cells with glycoprotein ligands on endothelial cells [78]. This process requires both sphingolipids as intermediates of the de novo sphingolipid biosynthesis pathway and sphingolipids as intermediates of the SM catabolic pathway (Figure 2).

#### 3.1.1. Sphingolipids as Intermediates of the De Novo Biosynthesis Pathway

Inhibition of sphingolipid de novo synthesis in THP-1 monocytes reduces their migration toward MCP-1 (monocyte chemoattractant protein 1). This could be achieved by knockdown of either serine palmitoyltransferase subunit 1 or partitioning defective protein 3 (Par3) in these cells [79]. In CerS2 knockout mice, migration of neutrophils is impaired that is possibly related to reduced production of very long chain glycosphingolipids and a reduced G-CSF expression as well as Lyn signaling in these mice [80]. Glycosphingolipids on human myeloid cells stabilize the binding of these cells to E-selectin [81, 82]. Downregulation of GluCer synthase (UGCG) in HL-60 cells reduced rolling of HL-60 on E-selectin but not on P-selectin bearing human umbilical vein endothelial cells (HUVEC). This leads to a reduced cell transmigration of UGCG-downregulated HL-60 cells across a HUVEC monolayer [83]. Also, Iwabuchi et al. have shown that migration of human neutrophils depends on LacCer at the plasma membrane [84]. Binding of a specific anti-Lac-Cer antibody (T5A7) to neutrophils induces migration. This is likely due to the activation of Src-family kinase Lyn and phosphoinositol 3 kinase (PI3K). But possibly also a G*α*- (i/o) coupled receptor is involved [84]. Especially in this work, it was demonstrated that there are distinct differences between human and mouse neutrophils. They detected a ∼20-fold lower LacCer content in plasma membranes in mouse than in human neutrophils [84]. Furthermore, the immune system of mice differs fundamentally from humans. For instance, in humans, neutrophils constitute with approximately 50–70% of the major population of circulating leukocytes, whereas in mice, neutrophils represent only 5–10% of blood leukocytes [85]. Therefore, comparing data generated in mice to human pathological conditions is critical, as the genetic or chemical-induced mouse disease models are only in part comparable to the situation in humans.

#### 3.1.2. Sphingolipids as Intermediates of the SM Catabolic Pathway

Treatment of neutrophils with the chemotaxin, formylmethionylleucylphenylanaline, leads to a translocation of n-SMase to plasma membranes where it is involved in the spreading and the extension of pseudopods. In these cells, n-SMase seems to influence the distribution of Rac 1/2 and RhoA to the leading edge of migration as this polarized distribution is totally lost when n-SMase was inhibited [86]. In line with these findings, factors associated with n-SMase activity-deficient leukocytes show also a disrupted chemotactic response. They protrude pseudopodia in all directions instead of having one clear leading edge, indicating that these cells are impaired in their navigation capacity to chemokines [87]. a-SMase is involved in mast cell migration [88].

In CerK-deficient macrophages, the MCP-1/CCR2 signaling pathway is attenuated implicating that C1P plays a role in macrophage migration [89]. Incubation of macrophages with C1P stimulates cell migration in a G(i) protein-dependent manner that causes phosphorylation of extracellularly regulated kinases (ERK) 1 and 2, protein kinase B, and activation of phospholipase C-*β*2 (PLC-*β*2) [90, 91]. Also, metalloproteinase- (MMP-) 2 and MMP-9 are upregulated in a PI3K and ERK 1/2- dependent manner after stimulation of macrophages with C1P [92]. Further studies show that C1P induces the release of macrophage chemoattractant protein-1 (MCP-1/CCL2 (CC-chemokine ligand 2)), which binds to the CCR2 or CCR4 receptor and influences thereby monocyte migration [93]. The role of S1P in immune cell activation and migration is already summarized in another review within this special issue, to which we want to refer here [94].

### 3.2. Recognition of Exogenous Agents

Toll-like receptors (TLRs) together with Nod-like receptors (NLRs) belong to a group of receptors (pattern recognition receptors (PRRs)) that are able to indicate the presence of several pathogen-associated molecular patterns (PAMPs) to immune cells which enable them to distinguish foreign organisms such as viruses, bacteria, fungi, and parasites from host cells [95–97]. Many receptors, important for the immune response, are clustered in lipid rafts upon activation [98–100]. The regulation of T-cell receptor signaling occurs in lipid raft [101–103]. Nevertheless, complex sphingolipids can also act as direct recognition receptors for microorganisms. However, binding of pathogens to a single saccharide is only weak, but adherence to multiple saccharides, as they are observed in lipid rafts, is strong [104, 105]. Various TLRs exhibit a cholesterol or sphingolipid binding-like sequence in their transmembrane region, indicating that they directly interact with specific lipids associated with rafts in the membrane [106]. Increased virus uptake was related to an enhanced expression of CD150 in lipid rafts at the cell surface [107]. The binding of pathogens to its cellular recognition receptors involves both sphingolipids as intermediates of the de novo biosynthesis pathway and sphingolipids as intermediates of the SM catabolic pathway.

#### 3.2.1. Sphingolipids as Intermediates of the De Novo Biosynthesis Pathway

Knockout animals of the subunit 2 of the serine palmitoyltransferase and of SM-synthase 1 or SM-synthase 2 in macrophages influence TLR signaling by preventing its proper translocation to the plasma membrane [108–110]. In CerS2 knockout mice, we could demonstrate that these mice develop more severe colitis after dextran sodium salt (DSS) treatment than CerS2 WT mice. CerS2-ko mice show significant changes in several sphingolipids like a drop in very long-chain CerS/(dh)-CerS and an increase in long-chain CerS/(dh)-CerS These changes are associated with a loss of the tight junction protein ZO-1 in colon epithelial cells leading to weakened endogenous defense against the microbiome and an increase in several immune cells in the colon [111]. Blocking of the dihydroCer desaturase, leading to the accumulation of dihydroCer in cultured cells, inhibits the infection of cells with HIV-1 [112]. GluCer or LacCer form membrane microdomains for the recognition and phagocytosis of microorganism. Microorganisms bind to PRRs at dendritic cells which undergo a conformational change, resulting in the translocation of the receptors into LacCer-enriched platforms [113]. Additionally, it is has been shown that LacCer and complex glycosphingolipids of cellular membranes such as Gb3 and GM1 are direct binding structures for bacteria and viruses (like *Haemophilus influenza*, *Neisseria meningitidis*, and *Polyomavirus*) [114, 115]. Berenson et al. could show that binding of *E. coli* enterotoxin LT-IIc to glycosphingolipids requires the whole glycosphingolipid and that neither the oligosaccharide nor the Cer alone is sufficient for binding. Furthermore, they demonstrated that also the chain length of the glycosphingolipid is important for the binding of LT-IIc [116].

#### 3.2.2. Sphingolipids as Intermediates of the SM Catabolic Pathway

The activation of the TLR4 by *Helicobacter pylori* or lipopolysaccharide (LPS) is dependent on the activation of a-SMase and Cer formation [117]. CerS are necessary and sufficient to mediate TLR4 translocation to the plasma membrane in a Src-dependent manner [117]. Avota and coworkers demonstrated that binding of measles virus to pattern recognition receptor on DCs leads to an activation of a-SMase and enhanced virus uptake into DCs [107]. In fact, it induces SMase activity that subsequently increases Cer-rich membrane platforms and initiated intracellular signaling processes by clustering different receptors into these platforms. Also, infection of human epithelial cells by rhinoviruses is dependent on a-SMase activity, as pharmacological inhibition or genetic deficiency of a-SMase prevents this infection [99]. This group showed further that activation of a-SMase comprises its translocation from intracellular compartments onto the cell surface that takes place by a microtubule- and microfilament-dependent transport mechanism. Also, infection with *Pseudomonas aeruginosa*, *Staphylococcus aureus*, or *Neisseria gonorrhoeae* requires the activation of a-SMase and subsequently the formation of Cer-enriched membrane platforms [114, 118, 119]. Treatment of mice with the a-SMase inhibitor amitriptyline and antibiotics prevents lethal *Staphylococcus aureus*-induced sepsis and death [120]. This observation leads to a phase II randomised, double-blind, placebo-controlled trial investigating the a-SMase inhibitor amitriptyline in patients with cystic fibrosis. The amitriptyline-treated CF patients showed a significant increase in lung function and weight after treatment for 1–3 years in comparison to placebo-treated patients [120]. These data indicate that inhibition of a-SMase might be a new therapy option for patients with cystic fibrosis, who suffer from perpetual infections.

### 3.3. Activation/Differentiation of Immune Cells

After binding of microorganism, their toxins, or cytokines to extracellular receptors, the immune cells get activated and reprogrammed to distinct subtypes. This reprogramming is a cell type-specific process and includes metabolic changes, DNA rearrangements, and differentiation. Furthermore, it leads to the production and release of cytokines and chemokines by these cells. The inflammasomes are multimeric protein complexes in macrophages and neutrophils that are involved in the production of the proinflammatory cytokine IL-1*β* and activated after the binding of microbes to these cells [117]. Also, activation and differentiation of immune cells involve both sphingolipids of the de novo biosynthesis pathway and the sphingolipid metabolic pathway from SM catabolism.

#### 3.3.1. Sphingolipids as Intermediates of the De Novo Biosynthesis Pathway

In hepatocytes, overexpression of CerS6, which is responsible for the production of C16-Cer, leads to an elevated TNF-*α* secretion via the activation of the p38 mitogen-activated protein kinase (MAPK) [121]. In line with these data, Ali et al. observed an enhanced activity of the TNF-*α*-converting enzyme (TACE) after the treatment of CerS2 knockout mice with LPS [122]. This results in elevated TNF-*α* level and worsens the outcome of LPS-induced septic shock in CerS2-ko mice. CerS2 knockout mice show also an upregulation of C16-Cer as a compensation mechanism to the loss of C24:0- and C24:1-Cer [123]. The activation of inflammasomes resulting in the release of IL-1*β* in macrophages seems to be independent from the sphingolipid de novo synthesis [124].

#### 3.3.2. Roles of Sphingolipids as Intermediates of the SM Catabolic Pathway

Both in the Cftr-deficient mice (mouse model for cystic fibrosis) and in the high-fat diet- (HFD-) induced glomerular injury mouse model, the activation of aSMase is associated with enhanced activity of inflammasomes. Knockout of a-SMase or caspase 1 inhibition protected Cftr-deficient mice from lung inflammation and kidney from HFD-induced injury [123]. Furthermore, knockdown of a-SMase in both mouse models prevents the production and release of IL-1. These data indicate that the activation of the a-SMase is an essential event in the activation of inflammasomes and subsequent production of proinflammatory cytokines. To which extent the generation of CerS by the aSMase itself is important for the formation of the inflammasome is not known as very recently it has been shown that activation of the S1PR1 contributes to the expression of NLRP3 inflammasome. As mentioned above, all sphingolipids are metabolically interconnected; therefore, CerS generated by the a-SMase are subsequently degraded by the ceramidase to sphingosine which can be phosphorylated to S1P that subsequently can activate different receptors. Weichand et al. have shown that the knockdown of the S1PR1 in tumor-associated macrophages leads to a reduced NLRP3 3expression and reduced IL-1*β* levels [125], indicating that S1P might be the important player in the activation of the inflammasome. However, Wang et al. demonstrated that loss of acid ceramidase 3 (Acer3), leading to an elevation in C18:1-Cer in blood mononuclear cells (BMCs), aggravates DSS-induced colitis, which is related to the hyperactivation of the innate immune system [126]. *In vitro*, Wang et al. could demonstrate that Acer3 deficiency enhanced and prolonged LPS-induced increases in the mRNA levels of IL-1*β*, IL-6, IL-23a, and TNF-*α* [126]. Activation of bone marrow-derived mast cells (BMMCs) by antigen/IgE leads to a 2.5-fold increase in a-SMase activity, an increase in [Ca^2+^]i, and the release of *β*-hexosaminidase. All these were impaired in antigen/IgE-stimulated a-SMase (−/−) BMMCs or by cotreatment with the a-SMase inhibitor, amitriptyline [88]. These data indicated that a-SMase-generated CerS are important for the activation of immune cells and the production of proinflammatory cytokines. CerK is also expressed in peripheral blood leukocytes. Here, it plays a role in phagocytosis and promotes phagolysosomal formation and fusion in polymorphonuclear leukocytes in a Ca^2+^-dependent manner [127, 128]. The degranulation of mast cells after binding to IgE is not only associated to an activation of aSMase but also positively influenced by the Ca^2+^-dependent CerK activation and consequent C1P production [16, 65]. C1P is also involved in the release of various proinflammatory prostanoids like PGE_2_ (prostaglandin E_2_) as the endogenous generation of C1P binds to the C2 domain of the cytosolic phospholipase A2*α* (cPLA2*α*) promoting thereby cPLA2*α* translocation to cellular membranes [129–131]. Interestingly, PGE_2_ can either promote or inhibit mast cell degranulation, dependent on the EP2/EP3 (E-prostanoid) receptor status of the cells [132]. This means that under some circumstances C1P might also inhibit mast cell degranulation. Unfortunately, all these mechanisms seem only slightly to be influenced in CerK^−/−^ mice [133], which calls the importance of C1P and CerK for mast cell function and eicosanoid synthesis into question. However, a detailed lipid analysis in these mice demonstrated that C1P levels are unchanged in the plasma of CerK^−/−^ mice [134], indicating that an adaptation mechanism takes place in these mice that compensates for the loss of CerK. However, Wijesinghe et al. already assumed that C1P subspecies especially not only d(18:1/18:0) but also C16:0 and C24:0 or C24:1 C1P are generated by alternative pathways besides CerK [135], but until now, it is not known how. Additionally, to intracellularly generated C1P, also, extracellular C1P influences immune cell activation. So the addition of C1P to LPS-activated neutrophils inhibits LPS-induced IL-8 production and NF*κ*B activation [136]. Also in vivo, in the LPS-induced acute lung injury mouse model, C1P attenuates the LPS-induced inflammation [136]. These data indicate that intracellular- and extracellular-generated C1P influences immune cells thereby rather leading to contrary effects. Binding of invaders to glycosphingolipids is important for entry into host cells by phagosomes and seems to prevent their fusion with lysosomes [137]. Sph is capable to inhibit phosphatidic acid phosphohydrolase in neutrophils [138] and the release of Ca^2+^ from endothelial cells [139]. Among SphKs, SphK1 is the isoform activated by proinflammatory cytokines [48] and plays an essential role in the TNF-*α*-triggered intracellular Ca^2+^ signal, degranulation, cytokine production, and activation of NF*κ*B, thus suggesting a pivotal role for SphK1 on the proinflammatory responses triggered by TNF-*α* [140, 141]. It is known that some of the effects of TNF-*α* are orchestrated by sphingolipid metabolites [142]. TNF-*α* stimulates the elevation of Cer and Sph, which has been shown to play a role in apoptosis in various cell types [143]. We found that Sph accumulates in the liver of mice treated with recombinant TNF-*α* [144]. The observed relationship between the toxicity of TNF-*α* mutants, the toxicity of Sph, and the extent of its accumulation in a murine liver provides evidence suggesting that Sph may be a mediator of TNF-*α*-induced cell damage and death [145]. TNF-*α* activates SM cycle during the induction of apoptosis [145]. Stimulation of HL60 cells with TPA (12-O-tetradecanoylphorbol-13-acetate) and simultaneous treatment with radioactive labelled serine leads to an increase in radiolabelled GluCer, LacCer, and GM3 in these cells after 48 h [146]. Receptors in LacCer-enriched platforms interact with the Src family kinase Lyn initiating the phagocytosis of the microorganism. The interaction between LynK and G protein can be influenced by the LacCer chain length thereby impacting the activation of neutrophils [113, 147].

## 4. Conclusions

In conclusion, when we investigate the role of a specific sphingolipid in physiological or pathophysiological processes, we have to keep in mind that sphingolipids are in a distinct equilibrium in the cell. Using mice, which bear a knockout for a specific gene of the sphingolipid pathway, the concentration of the sphingolipids depends on both the specific enzyme which is downregulated and various other sphingolipids which are a precursor or derivative of this sphingolipid. Several compensation mechanisms are induced by the accumulation of one specific sphingolipid, due to the knockdown of an enzyme using it as a substrate. This leads to an increase in sideways which also metabolizes this substrate leading to an upregulation of other sphingolipids. Therefore, we have to keep in mind that the observed effects in specific knockout mice might be related to the deregulation of various sphingolipids and/or the disturbance of an equilibrium and it is very likely that various sphingolipids together influence inflammatory processes.

## Abbreviations

a-SMase: Acid sphingomyelinase
alk-SMase: Alkaline sphingomyelinase
C1P: Ceramide 1-phosphate
Cer: Ceramide
CerK: Ceramide kinase
CerS: Ceramide synthases
CPTP: Ceramide-1-phosphate transfer protein
DAG: Diacylglycerol
GluCer-synthase: Glucosylceramide synthase
GluCer: Glucosylceramide
LacCer-synthase: Lactosylceramide synthase
LacCer: Lactosylceramide
LynK: Lyn kinase
n-SMase: Neutral sphingomyelinase
PAMPs: Pathogen-associated molecular patterns
PC: Phosphatidylcholine
PPC: Phosphorylcholine
PRRs: Pattern recognition receptors
RSM-synthase: Reverse sphingomyelin synthase
S1P: Sphingosine-1-phosphate
S1PL: S1P lyase
SM: Sphingomyelin
SM: Synthase
SMase: Sphingomyelinase
Sph: Sphingosine
SphK: Sphingosine kinase
SphPh: Sphingosine phosphatase.
